# Mass Spectrometry Imaging

**DOI:** 10.5702/massspectrometry.A0102

**Published:** 2022-02-25

**Authors:** Shuichi Shimma

**Affiliations:** 1 Department of Biotechnology, Graduate School of Engineering, Osaka University, 2–1 Yamadaoka, Suita, Osaka 565–0871, Japan

**Keywords:** mass spectrometry imaging, lipids, proteins, pharmaceuticals, isotopes, instruments

## Abstract

Mass spectrometry imaging (MSI) is a technique for obtaining information on the distribution of various molecules by performing mass spectrometry directly on the sample surface. The applications range from small molecules such as lipids to large molecules such as proteins. It is also possible to detect pharmaceuticals and elemental isotopes in interstellar matter. This review will introduce various applications of MSI with examples.

## 1. INTRODUCTION

The development of analytical technology in the history of science follows a particular trajectory. For example, the discovery of X-rays, which are electromagnetic waves, prompted the development of two-dimensional (2D) radiographs, followed by three-dimensional multi-slice X-ray tomography. In addition, 2D nuclear magnetic resonance (NMR), which originated from the Zeeman effect, was developed and employed in the medical field. In other words, analytical techniques originated from discoveries in physics, and were later extended from graph data to two- and three-dimensional image data.^1)^ In mass spectrometry, it is possible to obtain mass spectra from the difference in the motion of ions in an electromagnetic field. Mass spectrometry imaging (MSI) is a technique for visualizing distribution information of atoms and molecules on a sample surface using mass spectra. In the past, MSI was used to analyze the surface of inorganic materials using secondary ion mass spectrometry (SIMS). On the other hand, in the 1990s, there were reports^2)^ on the imaging of low molecular weight materials on the surface of living tissues by SIMS and on the MSI of proteins by matrix-assisted laser desorption ionization (MALDI). MSI research of biomolecules has rapidly developed since then.^3,4)^

In this review, I highlight the methodology and applications of MSI. First, the methodology of MSI is outlined, followed by a discussion of the instrumentation. Then, I review the applications of MSI in biology, medicine, food analysis, and isotope ratio analysis as examples of its applications in various fields.

## 2. INTRODUCTION TO MASS SPECTROMETRY IMAGING

The basic principle of MSI is direct mass spectrometry on the sample surface (*e.g.*, biological tissue surface). Commonly used ionization methods are MALDI and SIMS, as mentioned previously. In recent years, atmospheric pressure ion sources such as desorption electrospray ionization (DESI) have also been used. Since these ionization techniques ionize a single point on the tissue surface, the measurement is usually considered to be zero-dimensional. However, the information obtained by MSI is image information, *i.e.*, two-dimensional. To obtain a surface from a point, the following two methods can be considered.

### (1) Scanning MSI

**Figure 1:**
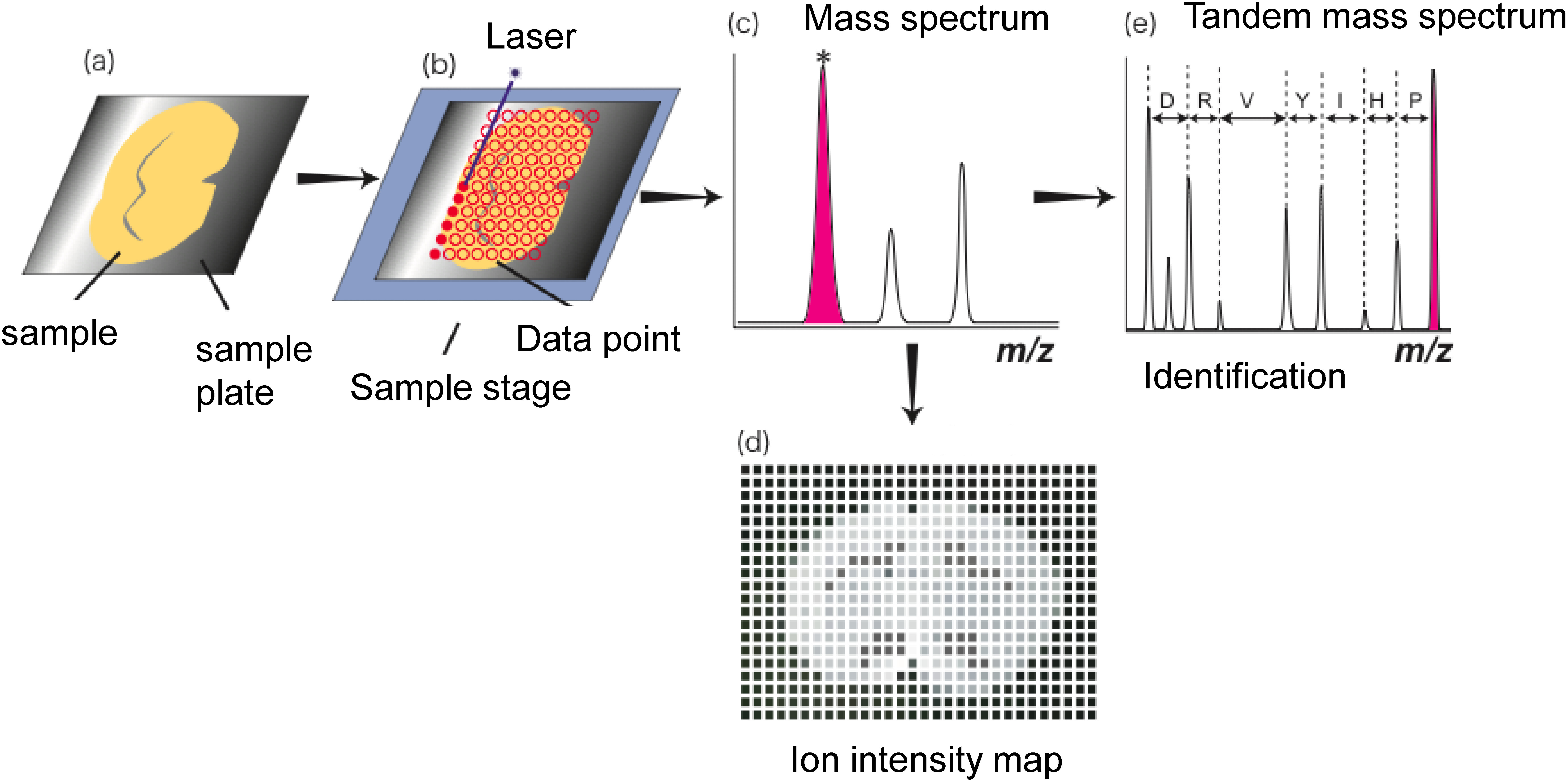
Fig. 1. Outline of scanning MSI. (a) Pretreated sample placed on a conductive material. (b) Mass spectra acquired at each data point after setting the laser irradiation points. (c) Peak of interest selected from the obtained spectrum. (d) Intensity distribution of the selected peak. (e) Molecule identified on the tissue surface by tandem mass spectrometry.

In scanning MSI (hereinafter referred to as the “scanning type”), *m* measurement points on the sample surface in the *x* direction and *n* points in the *y* plane are prepared, and acquire *m*×*n* mass spectra^5)^ (Fig. 1). The mass spectra automatically acquired at each point on the sample surface are stored with the position information. From the obtained mass spectrum, the intensity distribution at a certain *m*/*z* is captured. In this way, the automatic measurement function of a scanning type instrument can be used as it is. Therefore, in general, the term “MSI” primarily refers to the scanning type. Structural analysis is also possible by performing tandem mass spectrometry directly on the sample surface for ions with the *m*/*z* of interest.

In the scanning type, the spatial resolution of the image depends on the diameter of the irradiated spot and the spot spacing. When the spacing is smaller than the spot diameter, it is called “oversampling.” In the case of SIMS, the primary ion beam diameter is <100 nm and the image with high spatial resolution of submicron can be obtained. However, fragmentation occurs during ionization, and fragment ion imaging, especially of phosphocholine (fragment ion of phosphatidylcholine), is often observed. SIMS using argon and water clusters is now available, and the concept of “SIMS = many fragment ions” is changing.^6)^ On the other hand, in the case of MALDI, the laser spot diameter is approximately several μm to 100 μm, which limits the spatial resolution that can be obtained. On the other hand, MALDI is characterized by its ability to ionize both low and high molecular weight molecules.

In order to obtain a high spatial resolution image by scanning, it is necessary to acquire a large number of mass spectra. For example, if the resolution is doubled, *i.e.*, the data acquisition interval is halved, the number of data is quadrupled, and the measurement time becomes a problem. Notably, the measurement time is the product of the number of data points in the region of interests (ROIs) and the data acquisition time at each point. Here I consider to perform imaging of whole-body sagittal sections in 10-week-old rats. Under the measurement conditions shown in Table 1, it would take more than 10 h.^7)^ Thus, the measurement time varies from <1 h to several tens of hours, depending on the characteristics of mass spectrometers used and the spatial resolution required.

**Table 1:** Table 1. Example of scanning imaging mass spectrometry measurement conditions.

Size of ROI	16 cm×4 cm
Data acquisition interval	250 μm
Laser repetition rate	200 Hz
Number of laser beams	100 times/point

**Figure 2:**
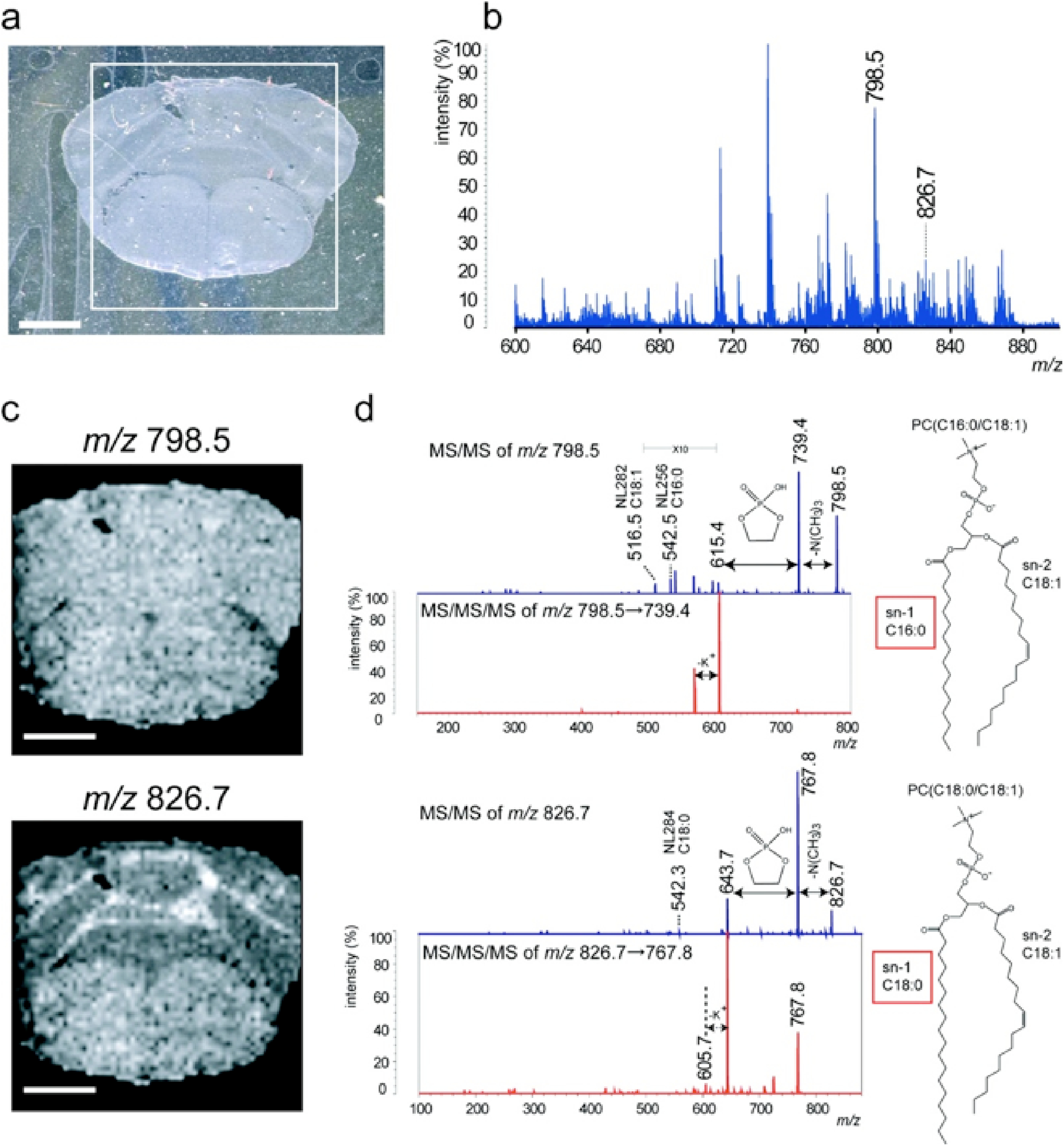
Fig. 2. Mouse cerebellar MSI. (a) Optical images of mouse cerebellar coronal sections. Squares represent imaging regions. (b) Averaged mass spectrum. (c) Imaging at *m*/*z* 798.5 and *m*/*z* 826.7. (d) Tandem mass spectra at *m*/*z* 798.5 and *m*/*z* 826.7; *m*/*z* 798.5 is phosphatidylcholine (PC) (C16 : 0, C18 : 1) and *m*/*z* 826.7 is PC (C18 : 0, C18 : 1). Scale bar: 1.5 mm (reproduced with permission from Ref. 5. ©American Chemical Society, 2008).

Figure 2 shows the imaging results of a coronal section of mouse cerebellum as an example of scanning measurement. Figure 2(a) is an optical image of the sample surface, and the square is the imaging region. Figure 2(b) shows the averaged mass spectrum obtained in the region, and Fig. 2(c) shows the intensity distributions at *m*/*z* 798.5 and *m*/*z* 826.7. Figure 2(d) shows the results of the tandem mass spectra of ions at *m*/*z* 798.5 and *m*/*z* 826.7 obtained on the tissue surface, which show that the structure of the carbon chain at position 1 is different in phosphatidylcholine, as shown in the figures, respectively.

### (2) Stigmatic MSI

**Figure 3:**
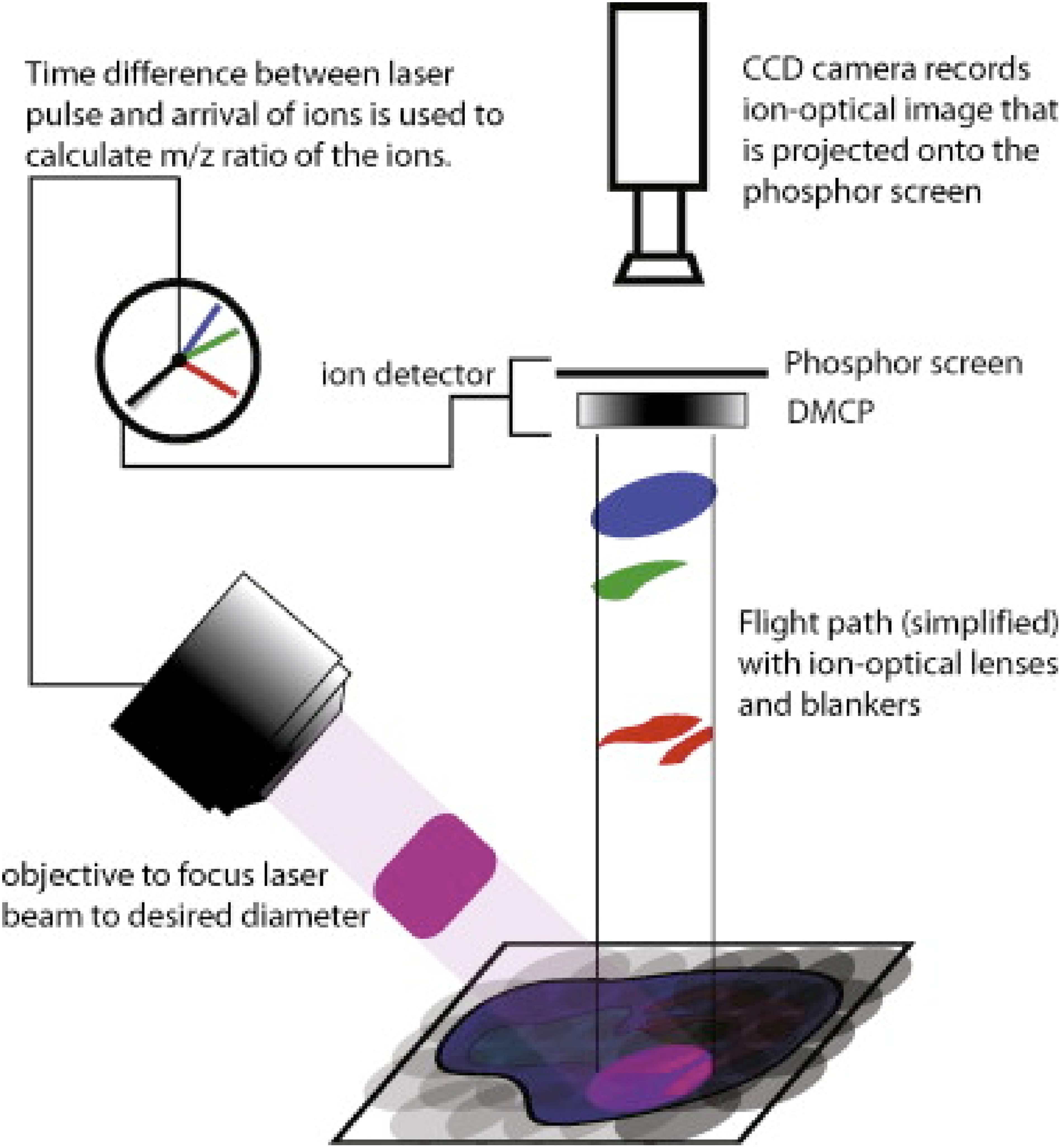
Fig. 3. Outline of stigmatic type MSI. In the case of MALDI, the laser diameter is widened, and in the case of SIMS, the ion beam diameter is widened to irradiate the tissue surface. (Reproduced with permission from Ref. 7. ©Elsevier B.V., 2009).

In the stigmatic type MSI (hereinafter referred to as the “stigmatic type”), ionization is performed, not as a point, but as a surface. In other words, a uniformly spread laser beam is irradiated to the sample surface in MALDI, and a uniformly spread primary ion beam is irradiated to the sample surface in SIMS. This method is referred to as the “stigmatic type” in contrast to the scanning type (Fig. 3). The irradiation area is ∼100–200 μm in diameter. As shown in Fig. 3, in the stigmatic type, the ionized ions arrive at the detector maintaining their distribution on the tissue surface. Therefore, a high-resolution image can be obtained using a detector with high spatial resolution. The detector can be a microchannel plate (MCP) combined with a fluorescent surface and a CCD camera, or an MCP combined with a 2D position sensitive device. In addition, it is also possible to detect the image as a magnification changeable image by arranging an ion optical system in front of the detector. In SIMS, a stigmatic type system combined with a magnetic field mass spectrometer has been employed, and a MALDI-TOF system has been developed.^8–10)^ In addition to the development of mass spectrometers mentioned above, detectors combining MCPs and delay line anodes have been developed to obtain high spatial resolution.^11,12)^ Stacked CMOS active pixel sensors (SCAPS)^13)^ and silicon on insulator (SOI) detectors have been developed to obtain high spatial resolution.

**Figure 4:**
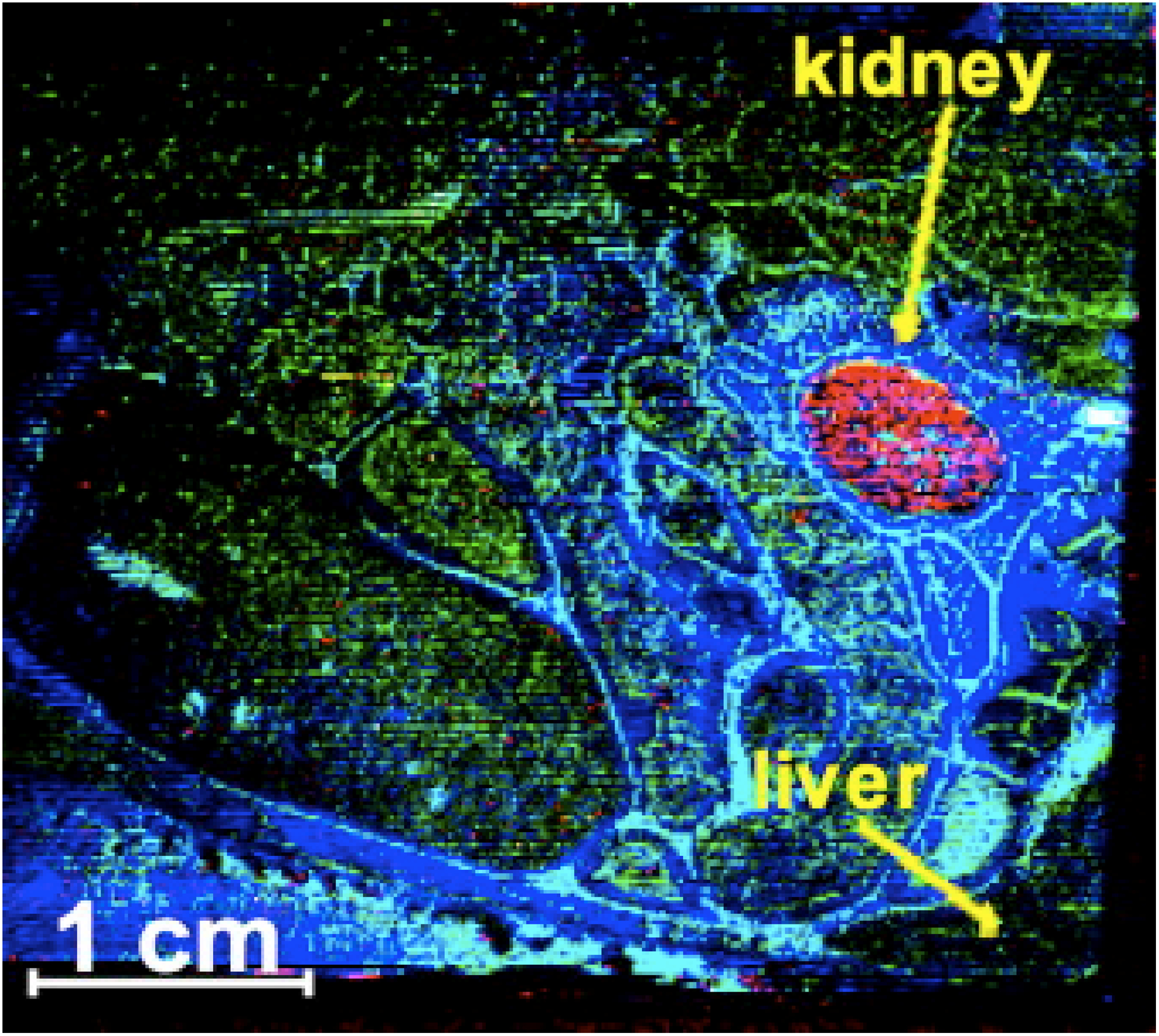
Fig. 4. Example of stigmatic MSI. The sample is a part of a whole-body sagittal section of the rat. Low molecular weight substances are denoted by blue; free choline (*m*/*z* 104) is denoted by green; phosphocholine (*m*/*z* 184) is denoted by red; metabolites (*m*/*z* 224). Phosphocholine is accumulated between organs and metabolites are accumulated in the kidney. (Reproduced with permission from Ref. 7. ©Elsevier B. V., 2009).

Figure 4 shows the imaging results of a whole-body sagittal section of a rat as an example of a stigmatic type measurement by MALDI.^10)^ The size of the ROI is 4 cm×4 cm, and 40,000 images are taken with a 200 μm diameter laser. In each image, a spatial resolution of less than 4 μm was obtained. In Fig. 4, the molecules of free choline (*m*/*z* 104), phosphocholine (*m*/*z* 184), and the metabolite of the drug are denoted by blue, green, and red, respectively. Metabolized drugs accumulate in the kidney and phosphocholine is localized between the organs. The high spatial resolution facilitates observation of the reticular distribution of each substance among the organs.

### (3) Characteristics of MSI

Compared with general imaging methods, such as optical microscopy, MSI has some distinct features.

Feature 1: A wide variety of molecular distributions can be obtained at a time.

One of the characteristics of mass spectrometry is the large number of molecular species that can be analyzed. Especially in the case of time-of-flight (TOF) MS, a wide mass range can be detected at once. This feature enables acquisition of the biodistribution of numerous phospholipid groups at a time or comprehensive visualization of the metabolites. Furthermore, it is possible to simultaneously detect phospholipids and peptides as well as drugs and their metabolites.

Feature 2: Ionizable objects can be visualized.

In MSI, the intensity distribution of ions detected by ionization on the sample surface is displayed. Therefore, once the ions are ionized, they can be visualized. This may appear normal, but it means that even a very small difference in *m*/*z* can be visualized as a different image if ionization is possible. As a result, it has recently become possible to present imaging results by peak separation with ultra-high mass resolution using a Fourier transform mass spectrometer or an ion mobility mass spectrometer.^14,15)^

Feature 3: Visualization is possible, even for substances that cannot be visualized by antibodies

The term “impossible to visualize by antibodies” has two meanings. First, it means that the antibody cannot be available. Second, it means that the structure cannot be recognized by antibodies. Nevertheless, MSI can visualize such molecules.

For example, the distribution of phosphatidylcholine according to its structure (Fig. 2) is a good example of these features. First, distributions are possible for all the peaks shown in Fig. 2(b) (Feature 1). Second, as shown in Fig. 2(c), different distributions are obtained for various structures of the carbon chain at position 1 of phosphatidylcholine. This is because “structural difference (excluding isomers)” is interpreted as “mass difference” in MSI (Feature 2). Furthermore, antibodies cannot recognize the structural differences of fatty acids because fatty acid moieties are usually located in lipid bilayers (Feature 3). Thus, lipid imaging (Feature 2) is attracting increased attention owing to its ability to utilize the features of MSI.

## 3. APPLICATIONS OF MASS SPECTROMETRY IMAGING

### 3.1 Biological Applications

#### Specific distribution of gangliosides in mouse hippocampus^16)^

**Figure 5:**
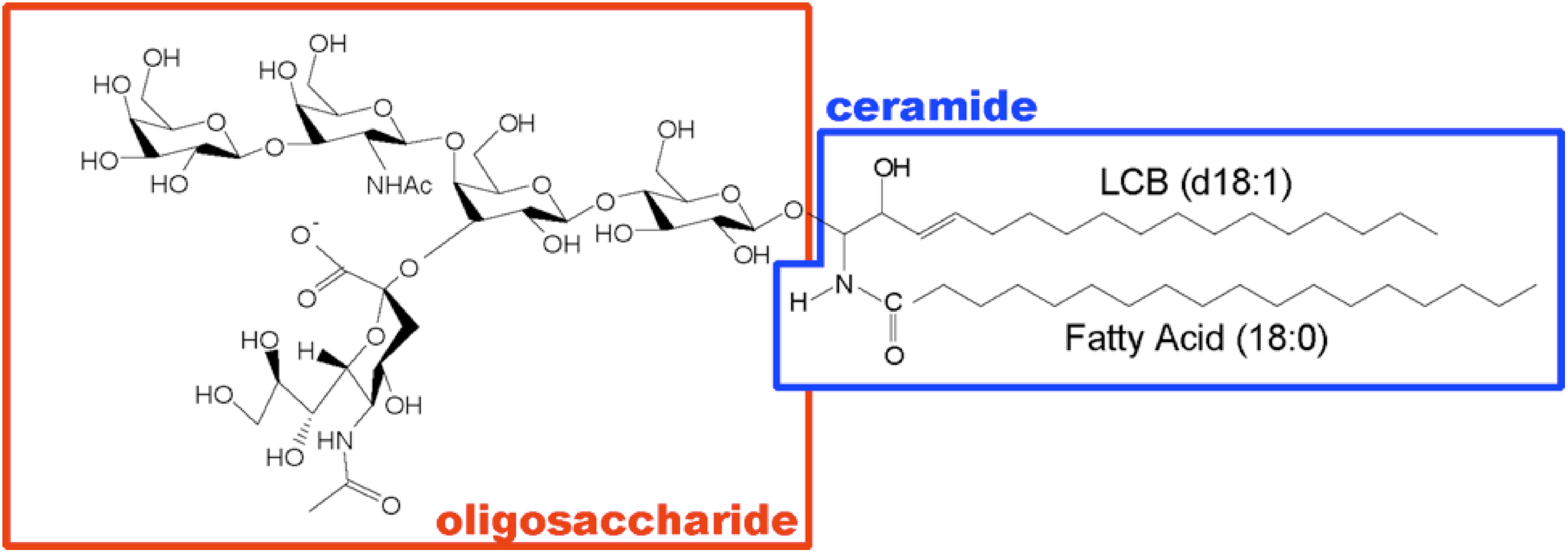
Fig. 5. Schematic diagram of ganglioside (GM1a). Gangliosides are one of the sphingoglycolipids and contain at least one acidic sugar, sialic acid, and the name of gangliosides varies depending on the number of sialic acids. Ceramide is composed of sphingosine (LCB) and fatty acid (FFA).

The hippocampus is a part of the limbic system and an organ involved in memory. The hippocampus has been studied because of its physiological importance as well as its characteristic anatomical structure. To date, imaging studies of the hippocampus have primarily been performed using confocal light microscopy, multiphoton excitation microscopy, transmission electron microscopy, or functional MRI (fMRI). In lipid-related studies, imaging of lipid rafts and glycosylation sites of glycolipids using antibodies has been performed, but imaging of structures in the cell membrane has not been performed. For example, Fig. 5 shows the structure of gangliosides, which are abundant in the central nervous system (CNS). Gangliosides can be divided into two parts, *i.e.*, the carbohydrate and ceramide moieties, which are outside and inside the cell membrane, respectively. Ceramides comprise sphingosine (long chain base (LCB)) and fatty acids. Gangliosides in the CNS are involved in memory formation, neurite outgrowth, synaptic transmission, and neuronal functions. It is impossible to distinguish differences in the ceramide structures inside the cell membrane using conventional visualization methods. Alternative methods have been used to extract, identify, and quantify the substances from a part of the tissue or a small area using laser microdissection. Therefore, it is difficult to know the distribution of the substance in the entire hippocampus.

**Figure 6:**
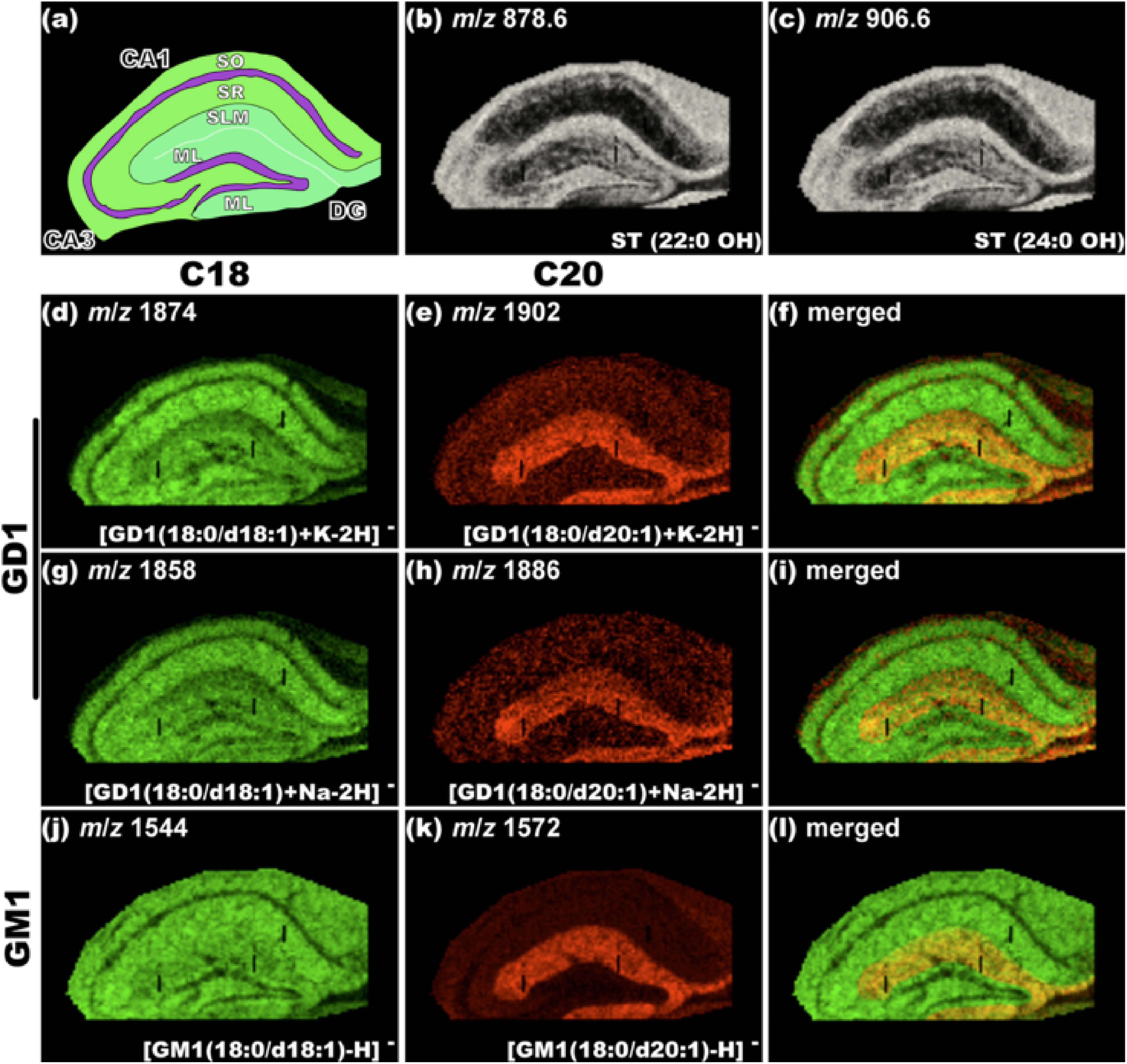
Fig. 6. Imaging results of gangliosides in the hippocampus. (a) Schematic diagram of the layered structure in the hippocampus. (SO; stratum ascending, SR; stratum radiatum, SLM; stratum reticulatum, ML; stratum molecularum) (b) and (c) Distribution of sulfatide, an acidic glycolipid (control). Potassium-added GD1 distribution. (d) C18 sphingosine. (e) C20 sphingosine. (f) Superposition of both. Sodium-added GD1 distribution. (g) C18 sphingosine. (h) C20 sphingosine. (i) Two-way superposition. Proton-added GM1 distribution. (j) C18 sphingosine. (k) C20 sphingosine. (l) Two-way superposition. (Reproduced with permission from Ref. 16. ©Plos One, 2008).

Figure 6 shows the imaging results of gangliosides in the mouse hippocampus. Figure 6(a) shows the schematic diagram of the imaging area, and Fig. 6(b, c) shows the distribution of sulfatide, an acidic glycolipid, which is high in white matter. Figures 6(d–l) show the distributions of GD1 (two sialic acids) and GM1 (one sialic acid), which are distinguished by the difference in the structure of sphingosine. In the figure, the distributions of sphingosine with 18 (C18 sphingosine) and 20 (C20 sphingosine) carbons are denoted by green and red, respectively. Notably, C20 sphingosine is localized in the stratum lacunosum-moleculare (SLM) and the lacunosum-moleculare (ML); this specific distribution was first shown by MSI.

**Figure 7:**
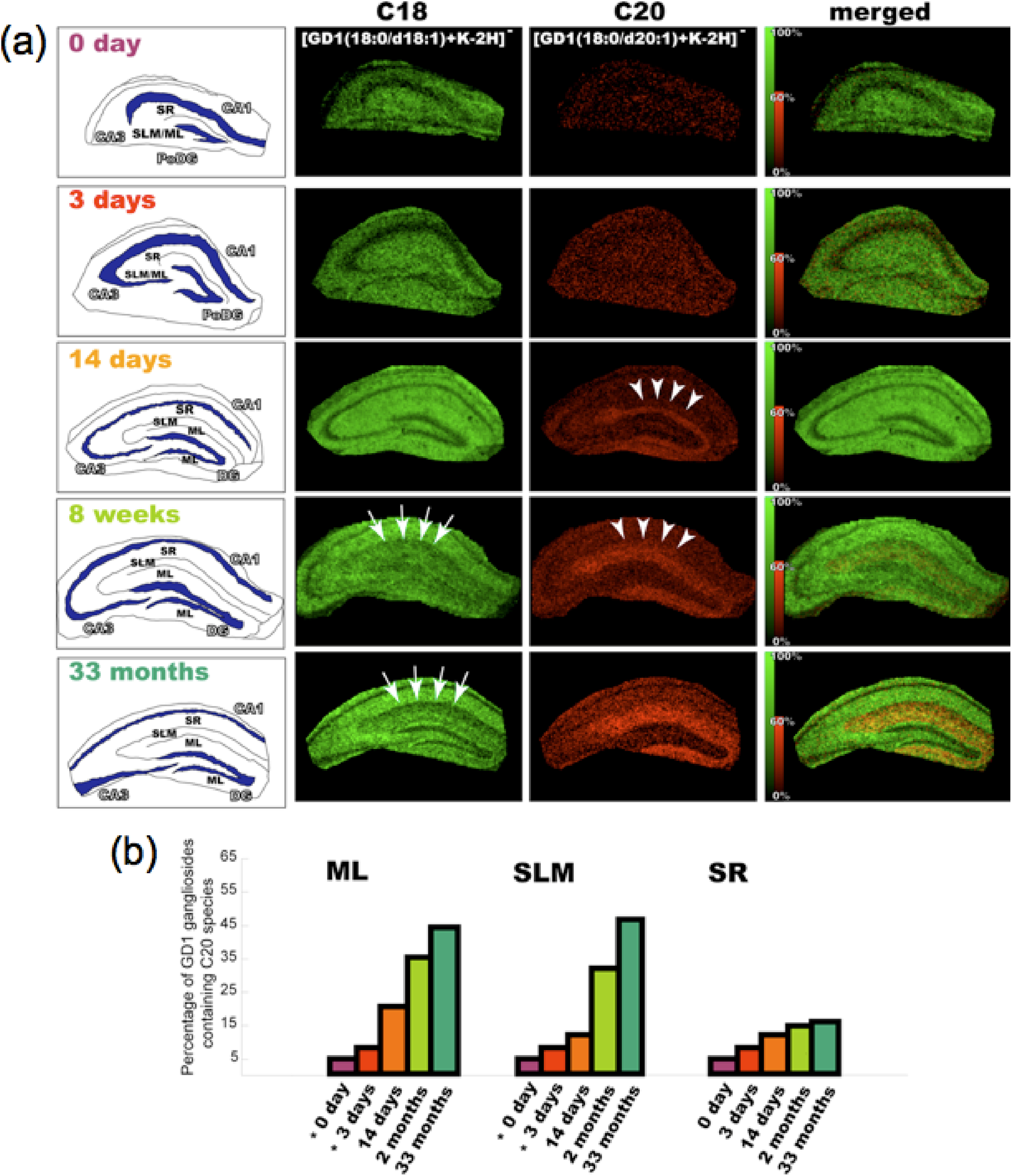
Fig. 7. (a) Age-related changes in the distribution of C18 and C20 sphingosine in GD1. Stratification is observed with aging. (b) Quantitative results of C20 sphingosine in different hippocampal regions. The accumulation of C20 sphingosine in the reticular molecular layer (SLM) is remarkable. (Reproduced with permission from Ref. 16. ©Plos One, 2008).

In addition, since biochemical methods have demonstrated that C20 sphingosine increases with age, we attempted to capture the change in its distribution by MSI (Fig. 7). GD1 imaging of mouse brain at 3 d, 14 d, 8 weeks, and 33 months after birth revealed specific accumulation of C20 sphingosine in ML and SLM at ∼14 d after birth. The accumulation was observed in the olfactory cortex and its projection sites. We speculate that the accumulation of C20 ganglioside increases the risk of neurodegenerative diseases such as Alzheimer’s disease, because this region is the site where selective neuronal loss is observed in early Alzheimer’s disease.

#### MSI of neuropeptides in crustaceans^17)^

**Figure 8:**
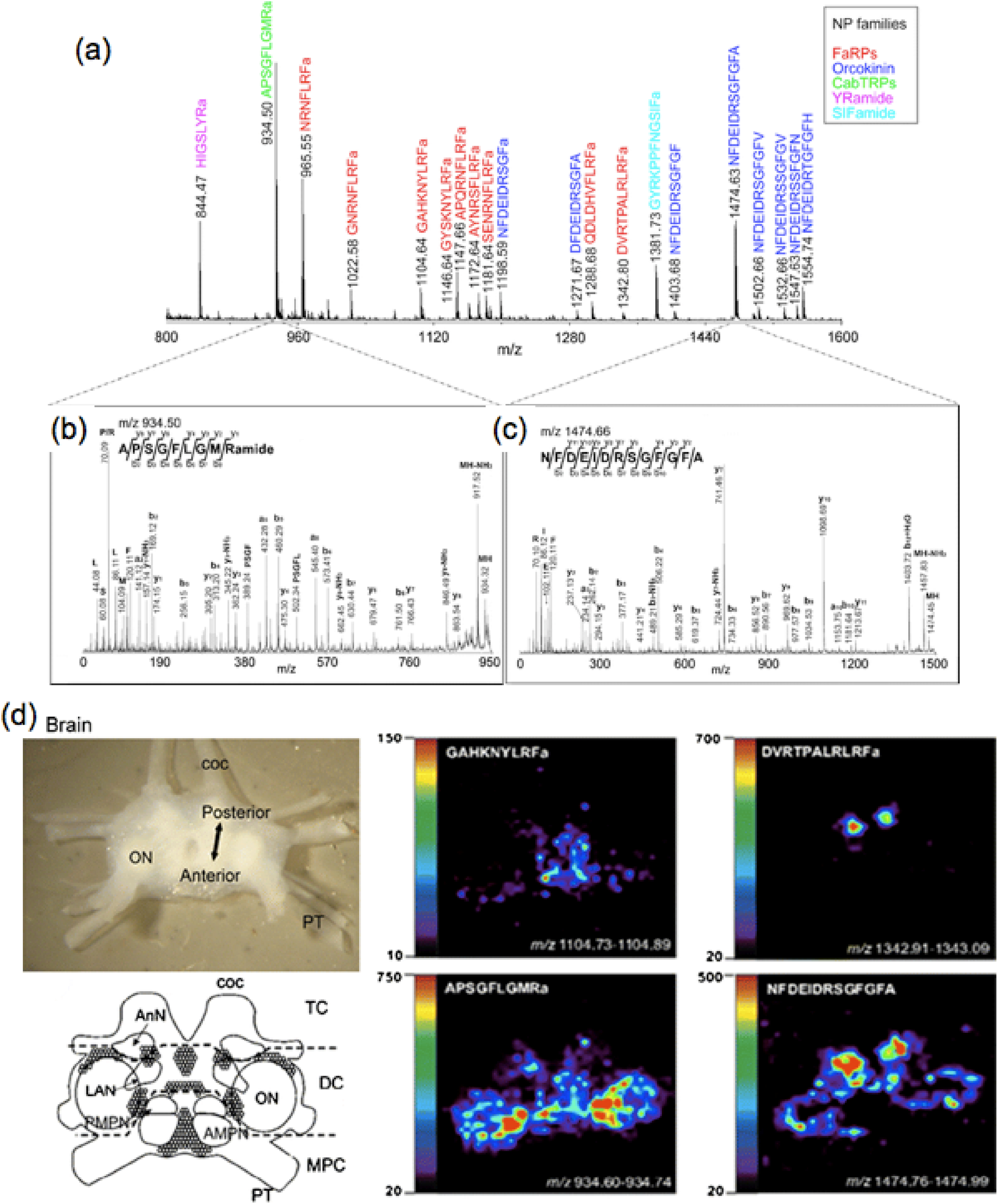
Fig. 8. Examples of neuropeptide imaging using *Cancer borealis* brain. (a) Mass spectrum obtained at the tissue surface. Each peak has been sequenced by tandem mass spectrometry. (b) Product ion spectrum at *m*/*z* 934 identified as APSGFLGMRamide from the peak pattern. (c) Product ion spectrum at *m*/*z* 1474. (c) Product ion spectrum at *m*/*z* 1474 identified as NFDEIDRSGFGFA from the peak pattern. (d) Neuropeptide imaging results. (Reproduced with permission from Ref. 17. ©Springer-Verlag, 2010).

Mollusks and crustaceans are often used in the study of biologically active substances. MSI is effective for the visualization of neuropeptides in mollusks and crustaceans because, as previously mentioned, it is possible to estimate a sequence of peptides that is unknown by structural analysis using tandem mass spectrometry on the tissue surface. Figure 8 shows an example of the neuropeptides identified in the brain of *Cancer borealis* (Jonah crabs) obtained with MALDI. Figure 8(a) shows the mass spectrum obtained from the tissue surface, and the identified amino acid sequences are shown above each peak. Figure 8(b, c) are the product ion spectra at *m*/*z* 934 and *m*/*z* 1474. Figure 8(d) depicts sample optical pictures and an example of imaging of the neuropeptides containing them. Figure 8(d) reveals that no method, other than MSI, can select a peak of interest (unknown substance) from the mass spectrum obtained on the tissue, identify the substance, then visualize the distribution of the substance. In Fig. 8(d), arginine-phenylalanine amide (RFamide: *m*/*z* 1104 and *m*/*z* 1342), tachykinin-related peptide (*m*/*z* 934), and orcokinin (*m*/*z* 1474) are shown in the brain. The localized sites in tachykinin-related peptides and orcokinin are different. RFamide is a cardiac stimulant, tachykinin is a smooth muscle contractor, and orcokinin is a physiologically active substance that controls the function of the anus and intestine.

#### Imaging mass spectrometry of *Bacillus subtilis* spores using nanoSIMS^18)^

**Figure 9:**
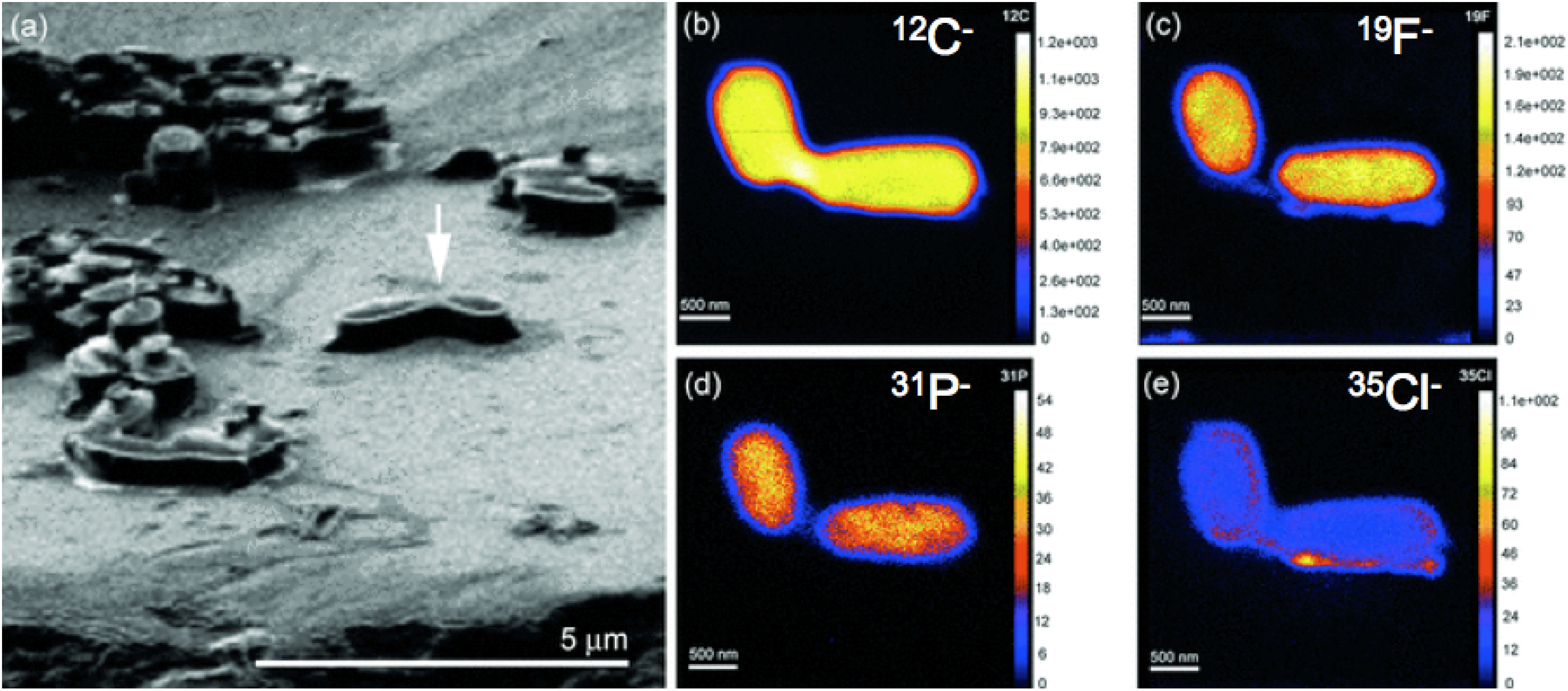
Fig. 9. Example of nanoSIMS imaging of *Bacillus thuringiensis* spores. (a) Scanning electron micrograph (arrow: spore captured). (b) ^12^C^−^ distribution. (c) ^19^F^−^ distribution. (d) ^31^P^−^ distribution. (e) ^35^Cl^−^ distribution. (Reproduced with permission from Ref. 18. ©The Royal Microscopical Society, 2009).

SIMS can image a small area of a few micrometers using a narrowly focused primary ion beam. Figure 9 shows an example of the imaging of spores of *Bacillus thuringiensis*, a *Bacillus subtilis*. Because the size of the spores is <5 μm, the sections are prepared by focused ion beam (FIB), which is also used to prepare samples for electron microscopy. In the example shown in Fig. 9, the thickness of the sample is 100–150 nm, and imaging of ^12^C^−^, ^19^F^−^, ^31^P^−^, and ^35^Cl^−^ is performed by scanning the fabricated sample with a primary ion beam focused at 50 nm. For each of them, characteristic distributions are observed in the microstructure. For example, carbon is uniformly distributed, but phosphorus is localized in the nucleus of the spore. This result is consistent with the fact that DNA and RNA are localized in the nucleus. On the other hand, for the halogens fluorine and chlorine, fluorine is localized inside the spores, while chlorine is localized on the outer surface. These halogens are presumably related to the germination of spores, but this has not yet been verified. Such new findings from the imaging results may lead to an understanding of the phenomenon, which has not been clarified to date.

### 3.2 Medical and Pharmaceutical Applications

#### Imaging of Cancer Tissue^19)^

MSI has been used to visualize various types of cancer tissues. The visualized cancers include brain tumors, lung cancer, liver cancer, colorectal cancer, and breast cancer. In this section, I explain the role of MSI in cancer research. First, as in conventional histological methods, MSI can be used as a new method to search for biomarkers by identifying the molecules contained in the tissue while observing its morphology. Second, if the biomarker is already known, it is expected to play a role in visualizing the distribution of the marker molecules and determining the extent of resection. For example, at present, pathological tissues are collected in the operating room or by biopsy, and the extent of resection is determined by staining the tissue and observing the condition. However, there is a possibility that invisible cancers may be left behind. Visualization at the molecular level may make it possible to reduce such fears in the future.

**Figure 10:**
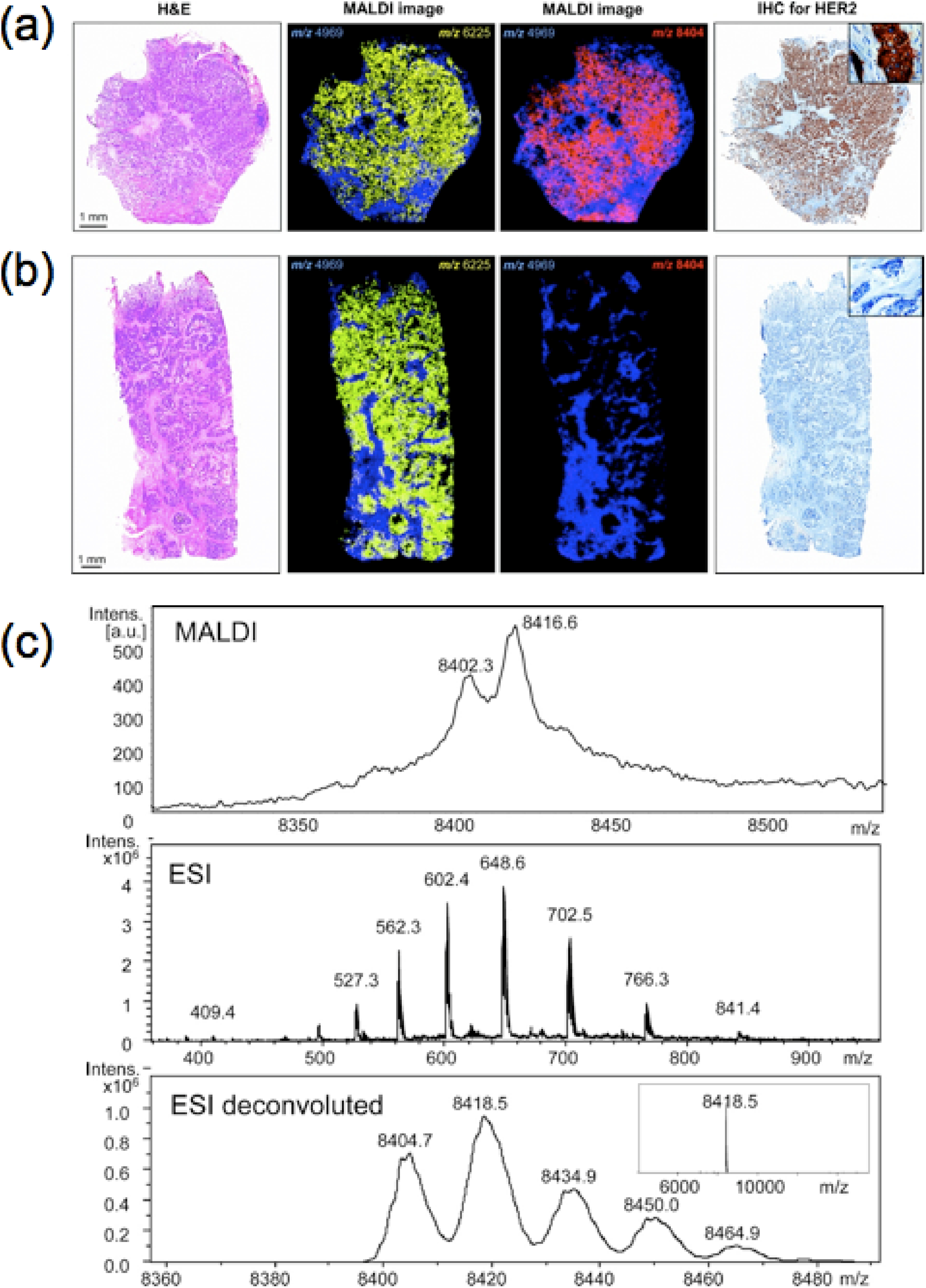
Fig. 10. Examples of protein imaging in breast cancer and non-cancer tissues. (a) HER2-positive tissue. (b) HER2-negative tissue. In each figure, hematoxylin-eosin staining (HE staining) is shown on the left and HER2 antibody immunostaining is shown on the right. In the imaging results, *m*/*z* 4969, *m*/*z* 6220, and *m*/*z* 8404 (CRIP1) are denoted by blue, yellow, and red, respectively. *m*/*z* 4969 is localized in the stroma, the *m*/*z* 6220 is non-specific in both tissues, and the *m*/*z* 8404 is specific to the cancerous area. (c) Comparison of mass spectrum by MALDI on the tissue and spectrum by ESI of the extract. (Reproduced with permission from Ref. 19. ©American Chemical Society, 2010).

Here, I focus on the MSI of proteins in breast cancer tissues. Figures 10(a) and 10(b) are examples of tissue staining and imaging results of breast cancer tissue and non-cancer tissue, respectively. In this report, statistical analysis was performed by imaging 30 individuals in the detection group and 18 individuals in the confirmation group. Among them, we found that seven types of peptides and proteins increased with overexpression of human epidermal growth factor receptor 2 (HER2). For example, peaks at *m*/*z* 8404 and *m*/*z* 4969, which is only localized in the stroma surrounding the tumor, are denoted by red and blue, respectively (Fig. 10). The peak at *m*/*z* 6225, which is always expressed regardless of HER2 positivity or negativity, *i.e.*, it is not a biomarker, is denoted by yellow.

The peak at *m*/*z* 8404 has been identified as CRIP1 (cysteine-rich protein 1). At present, tandem mass spectrometry is rarely used to identify the full-length amino acid sequence of such a large molecular weight protein directly on the tissue surface. The identification of amino acid sequences of proteins is generally performed by electrospray ionization (ESI) of multiply charged ions and collision-induced dissociation or electron transfer dissociation in an ion trap or Fourier transform mass spectrometer. The identification of CRIP1 is performed by extraction and purification of the protein by liquid chromatography (LC) and ESI-MS from the obtained fractions.

#### Pharmacokinetic analysis using MSI^20)^

In the drug discovery process, it is very important to know how the target compound is distributed *in vivo* when it is administered. In order to know the distribution of the administered compound in the body, the compound is radiolabeled (*e.g.*, ^3^H, ^14^C, and ^125^I as a β-ray source) and exposed to X-ray film, photographic dry plate, and imaging plate as a tracer, and the distribution is observed. These methods are termed autoradiography (ARG). In particular, the method is termed whole-body autoradiography (WBA) for the observation of whole-body pharmacokinetics and microautoradiography (MARG) for observation at the cellular level. ARG is a well-established technique that is still in use because it has the advantage of having a very high spatial resolution. However, there is a challenge in that the imaging time is long. In particular, the exposure time is several weeks to several months in the case of the photographic method using films, and 4–7 d in the case of the fluorescent method using imaging plates. Another problem in ARG is that it is impossible to distinguish between the administered compound and its metabolites. Currently, to prevent this problem in ARG, compounds are extracted from ARG images and their tissues, identified and quantified by LC-MS, and data from both methods are combined.

In pharmacokinetic imaging, MSI is attracting significant attention as an alternative method. This is because it has two advantages over existing methods. First, the drug can be imaged without having to perform radiolabeling of the compound. Since the structural information obtained by mass spectrometry and tandem mass spectrometry is substance-specific, it is possible to create distributions based on the mass of the administered compound or the masses of the expected metabolites. Second, the distributions of many compounds can be obtained from a single sample in a single measurement. This also leads to reduced time, which is a problem in existing methods. Thus, the development of a “label-free simultaneous detection” method in the future is expected to contribute to drug discovery research.

**Figure 11:**
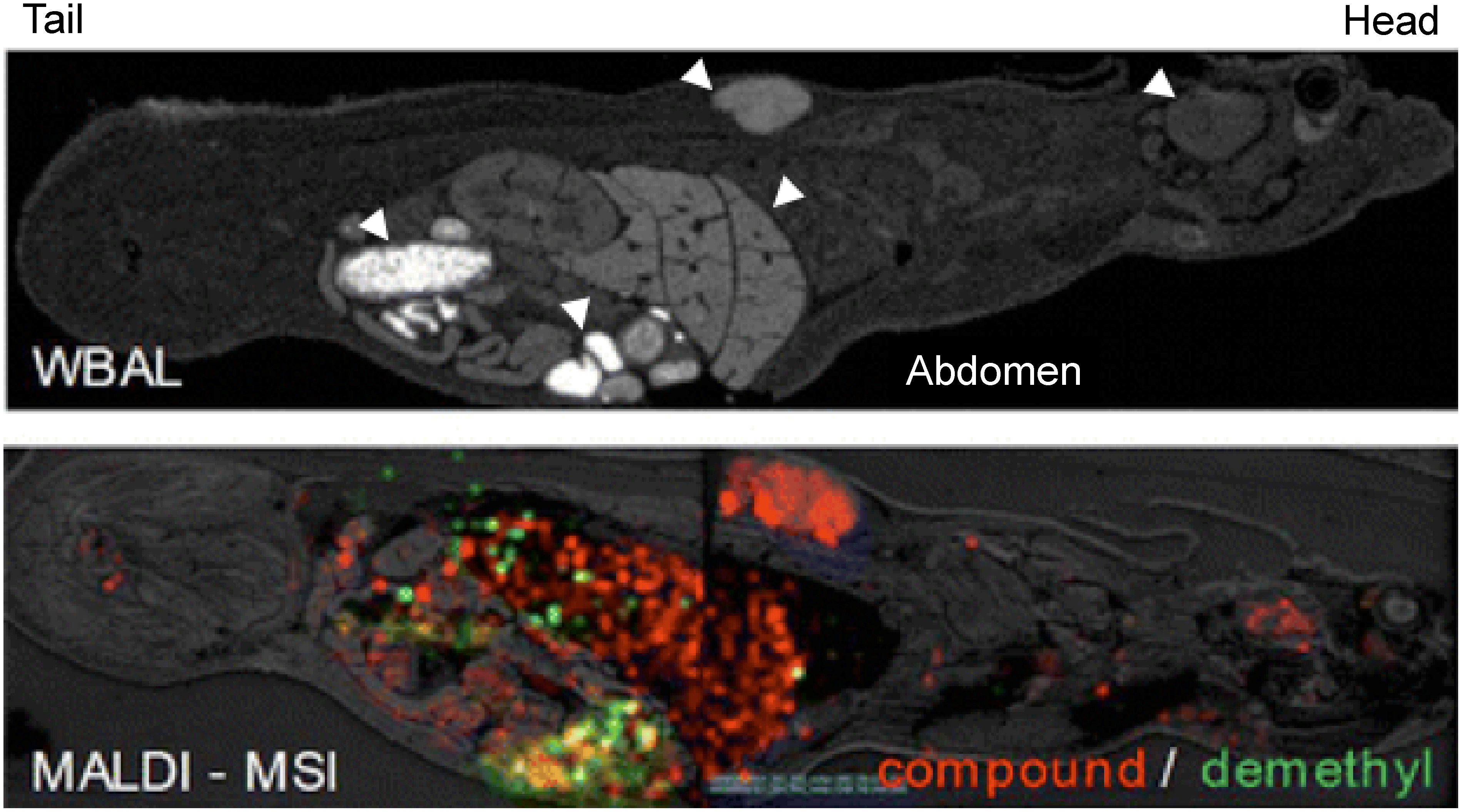
Fig. 11. Top: whole body autoradiography of rat. Bottom: whole body MSI of rat. Red: administered compound. Green: metabolites of administered compound. Co-localized sites are indicated in yellow. (Reproduced with permission from Ref. 20. ©American Association of Pharmaceutical Scientists, 2010).

Figure 11 shows the results of WBA and MALDI-MSI in rats. The upper panel of Fig. 11 shows the results of imaging by WBA and localization of the drug in the organs indicated by the arrowheads (brain, liver, bladder and so on). On the other hand, the MALDI-MSI results of the lower part of Fig. 11 show the same distribution as that of WBA. In this figure, it is important to note that the image distinguishes between the administered compound (red) and the metabolites (green), which have lost their methyl groups. In particular, the metabolites are localized in the abdomen.

As described above, MALDI-MSI of drugs *in vivo* has many advantages, but there are some points that need to be improved. One of them is that the matrix used in MALDI can be a contaminant peak of the drug-derived peak in the mass spectrum. To avoid this problem, the following methods have been proposed.

##### i. SIMS-based approach

In the SIMS ionization, the matrix is not necessary or is coated with metal, so that the effect of foreign peaks such as in MALDI is expected to be reduced. However, since SIMS is a hard ionization method, compounds are mainly detected as fragment ions. Recently, a SIMS method using cluster ions as the primary ion beam to realize soft ionization has been developed.

##### ii. Matrix-free or inorganic matrix-based approach

Currently, matrix-free ionization methods (*e.g.*, desorption ionization using through-hole alumina membrane (DIUTHAME))^21)^ and ionization methods using inorganic matrices that do not ionize themselves have been developed.^22)^ These techniques are expected to be applied to MSI because they remove the influence of contaminant substances in the low-molecular-weight region.

### 3.3 Application in food analysis

“Label-free,” “multi-component simultaneous detection,” and “specific visualization” are the features of MSI, which are not found in other imaging methods. In particular, in the case of MSI for low-molecular-weight compounds such as those discussed here, visualization can be performed using the raw sample without much pretreatment of the sample (in the case of MALDI, only the matrix is supplied). Therefore, there is an advantage in that the distribution information of the sample can be obtained as is. For example, in a previous report, the distribution of gamma-aminobutyric acid was visualized, an inhibitory neurotransmitter, in *Solanum melongena*, and showed that it was localized around the seed.^23)^

**Figure 12:**
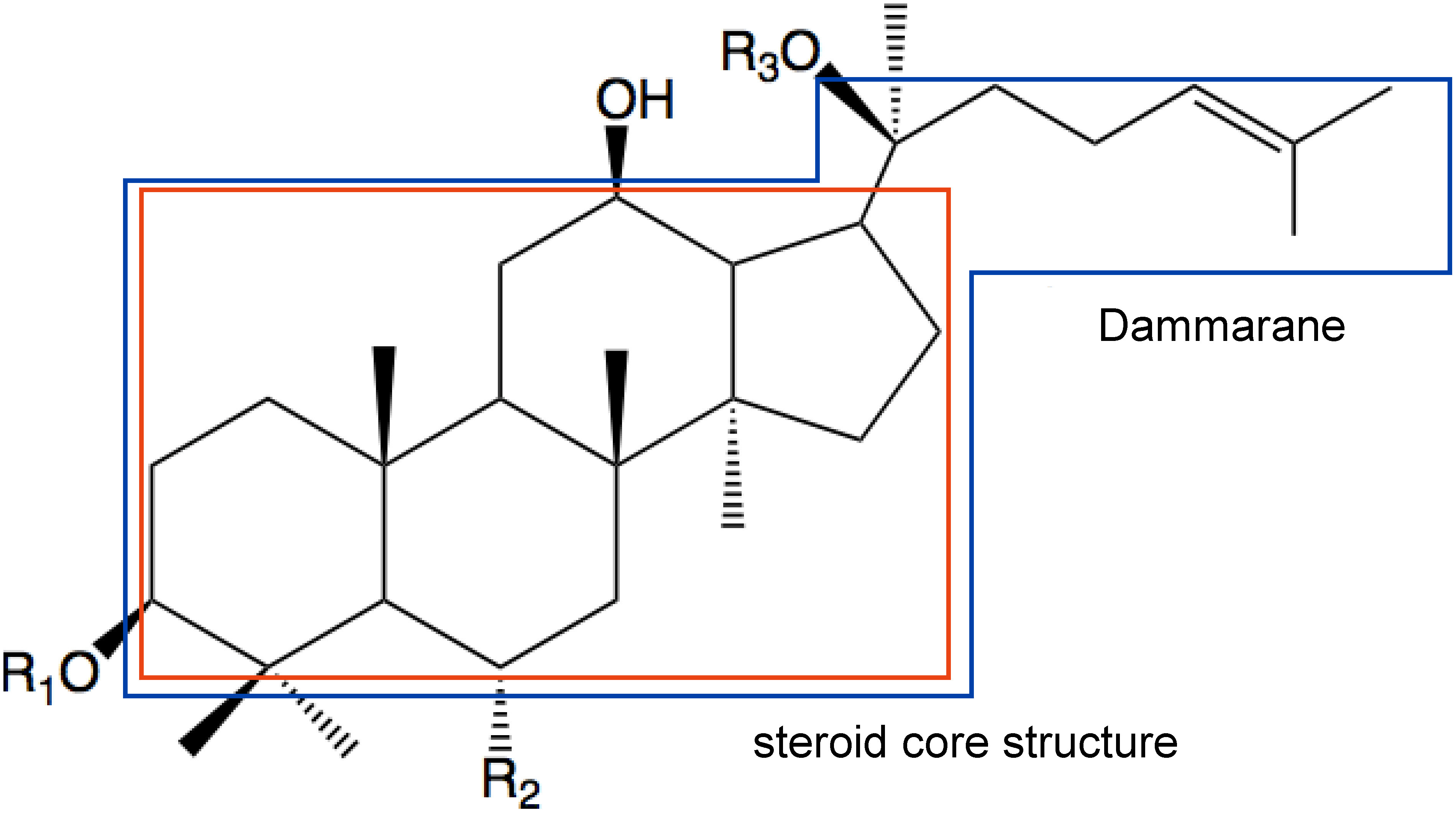
Fig. 12. Basic framework of ginsenosides. R1, R2, and R3 are functional groups to which sugar chains are attached.

**Figure 13:**
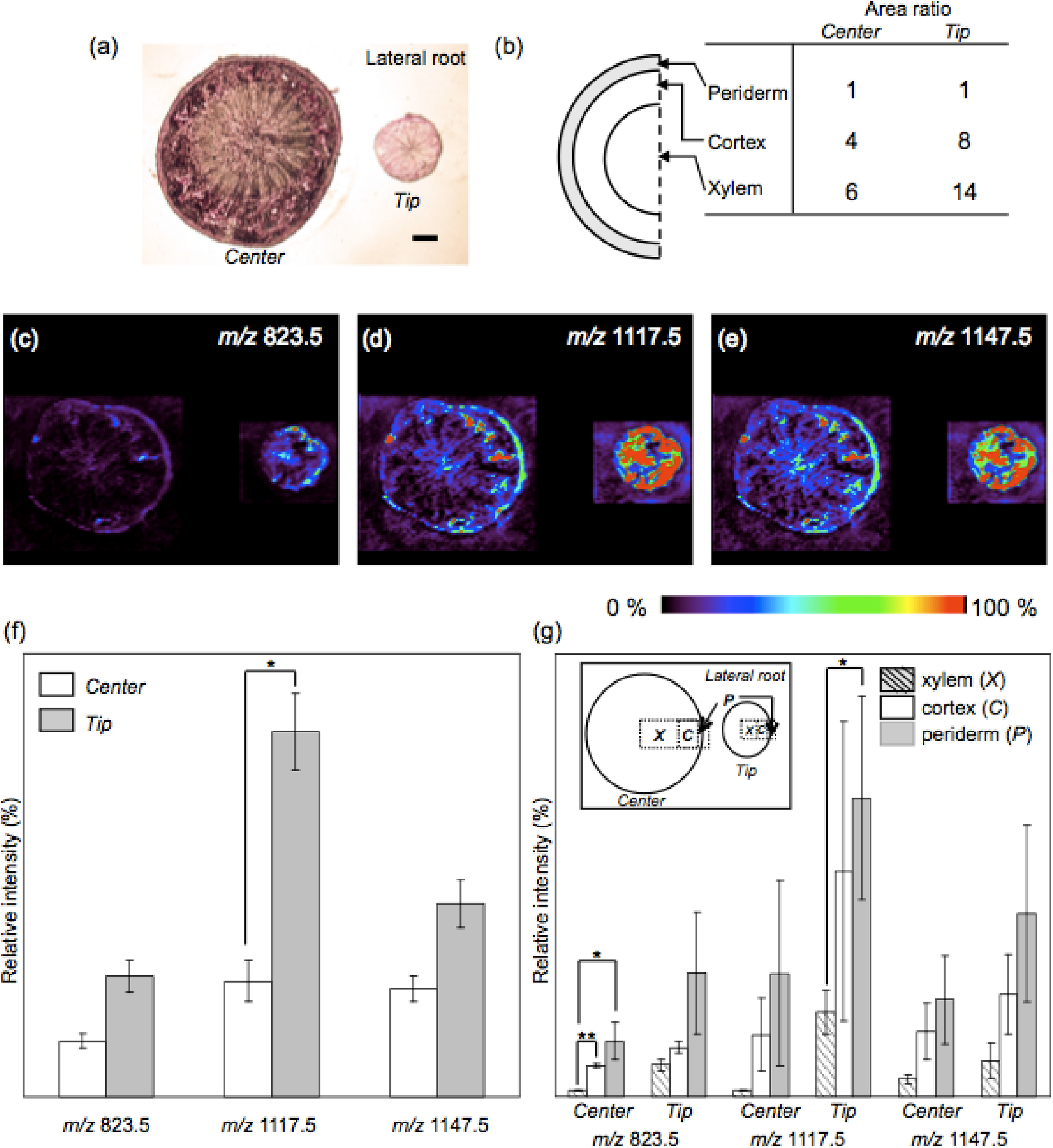
Fig. 13. Distribution of ginsenosides in ginseng. (a) Optical photograph of the sample. (b) Schematic diagram of the sample (periderm: pericarp, cortex: cortex layer, xylem: xylem). Imaging results (c) *m*/*z* 823.5, (d) *m*/*z* 117.5, (e) *m*/*z* 1147.5. (f), (g) Quantification results. (Reproduced with permission from Ref. 24. ©World Scientific Publishing Company, 2010).

There is also an example of the distribution of ginsenosides, an active ingredient of *Panax ginseng*.^24)^ Since monoclonal antibodies are available for ginsenosides, it is possible to visualize ginsenosides by immunostaining. For example, the anti-GRb1 antibody recognizes the Dammarane skeleton of ginsenosides shown in Fig. 12, which has a steroid core. Ginsenosides are a type of glycoside, and there are more than 30 types of ginsenosides depending on the structure of the aglycone (non-saccharide part of the glycoside) and the functional group (glycan) (R_1_, R_2_, and R_3_ in Fig. 12). Therefore, it is impossible to specifically visualize the distribution of the different structures of the side chains with antibodies that recognize the dammarane skeleton. Figure 13 shows the results of the visualization of the distribution of ginsenosides according to the differences in the functional groups by MSI. Figure 13(a) is an optical image of a sliced sample section. The difference in the distribution of the effective component between the center and tip of the specimen was visualized. Figure 13(b) shows the schematic diagram of the sample, which is composed of three layers (periderm, cortex, and xylem). Figure 13(c) shows the imaging results of the ginsenoside-derived peaks, *m*/*z* 823.5 (ginsenoside R_f_), *m*/*z* 1117.5 (ginsenoside R_b2_ or R_c_), and *m*/*z* 1147.5 (ginsenoside R_b1_). First, ginsenoside R_f_ (*m*/*z* 823.5) is mainly distributed in the periphery, and ginsenoside R_b2_, R_c_, and R_b1_ are localized in the periphery and in the stratum corneum. Next, the distribution of ginsenosides according to the difference in imaging site shows that all ginsenosides are localized in the apical region rather than the body. Figures 13(f) and 13(g) show semi-quantitatively that all ginsenosides are localized in the apical region. This application is the advantage of MSI is that it can visualize detailed structural differences, even for compounds for which monoclonal antibodies are available, such as ginsenosides.

**Figure 14:**
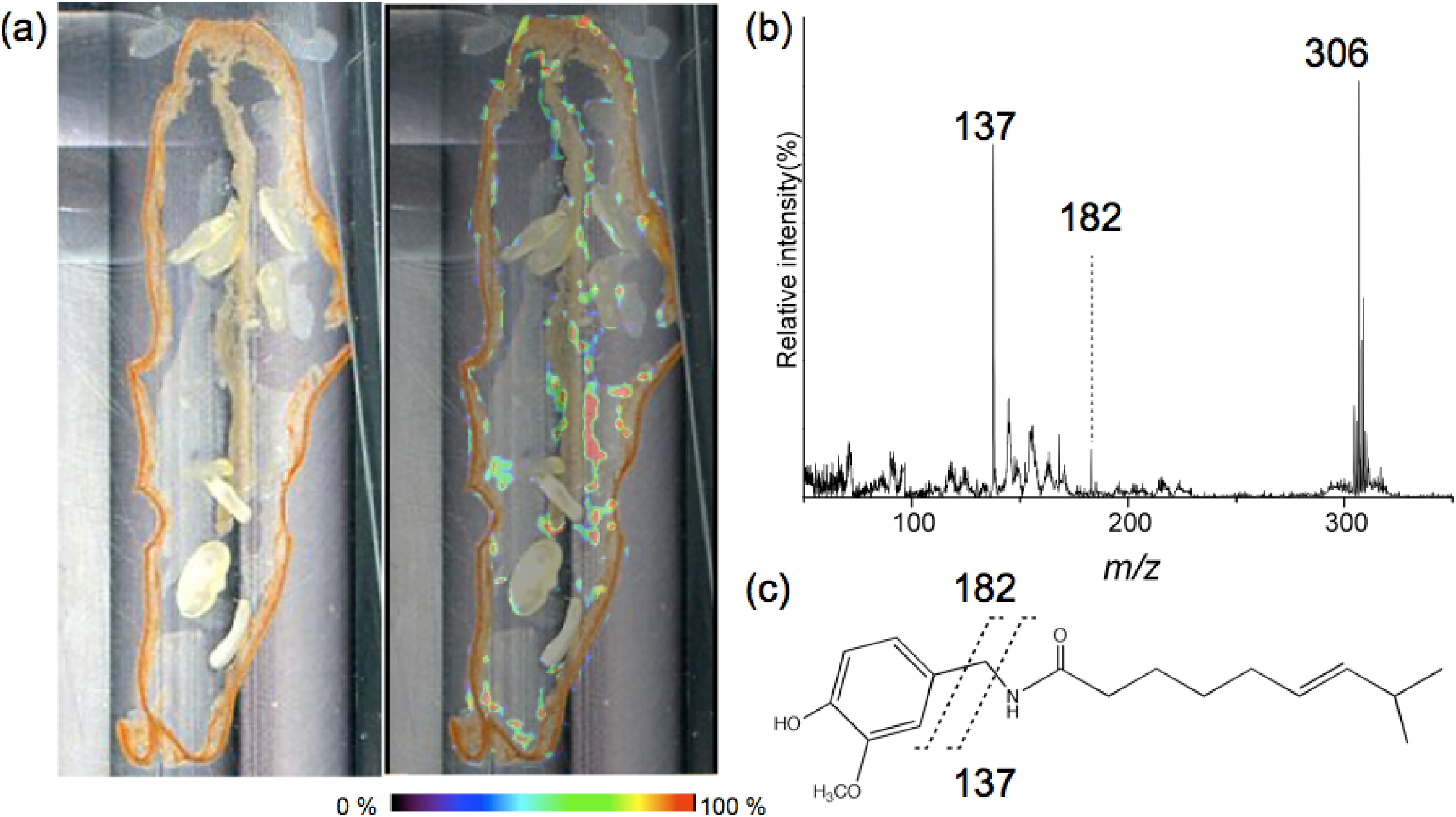
Fig. 14. Distribution of capsaicin in *Capsicum annuum*. (a) Sample photograph and capsaicin imaging result. (b) Product ion spectrum of capsaicin (*m*/*z* 306) obtained at the tissue surface. (c) Capsaicin structure and identification of product ion. (Reproduced with permission from Dr. Shu Taira, Fukushima Prefectural University).

Next, I discuss the example of capsaicin visualization in *Capsicum annuum*. To visualize capsaicin, it was radiated with ultraviolet light (280 nm). However, this method cannot be used for the imaging of capsaicin *in vivo* because many compounds with the same absorption wavelength are contained in the living body. In such a case, MSI is the most suitable method. Figure 14 shows the results of imaging capsaicin in a section of *Capsicum annuum*. Figure 14(a) depicts an optical photograph of the sample and the imaging result; based on this image, capsaicin is localized on the surface of the placenta (the part where the seed is attached). In addition, there is some capsaicin in the pericarp and almost none in the seeds. Figure 14(b) shows the product ion spectrum of the peak at *m*/*z* 306, which is expected to be derived from capsaicin. The peaks at *m*/*z* 137 and *m*/*z* 182 are the dissociation products at the site shown in Fig. 14(c). From this result, capsaicin is produced to protect themselves and seeds from external predators. Therefore, the abundant distribution in the pericarp and placenta is consistent with this argument.

As described above, MSI in food analysis is expected to be used in the future to visualize the distribution of functional secondary metabolites that cannot be identified by antibodies or for which antibodies do not exist.

### 3.4 Isotope Imaging

In this section, I discuss new isotope ratio studies using MSI. Pre-solar particles are particles with isotopic ratios different from the solar average. Pre-solar particles are particles with different isotopic ratios from the average value in the solar system, and they cannot be detected without isotope ratio analysis because they are mineralogically similar to minerals on earth. Both “scanning” and “stigmatic” SIMS are used for isotope imaging of pre-solar particles. In the field of space science, isotope imaging has been performed using scanning SIMS (*e.g.*, Cameca Nano-SIMS).^25)^ However, a long analysis time was required for high-precision quantitative imaging, and it was difficult to efficiently analyze a wide area due to time constraints. The newly developed isotope microscope, which is a stigmatic-type imaging system, enables us to perform quantitative imaging with high precision (per mil order) and high spatial resolution (submicron order) in a short time. Therefore, for the first time, various types of pre-solar particles from a large number of samples were successfully discovered.^26,27)^

**Figure 15:**
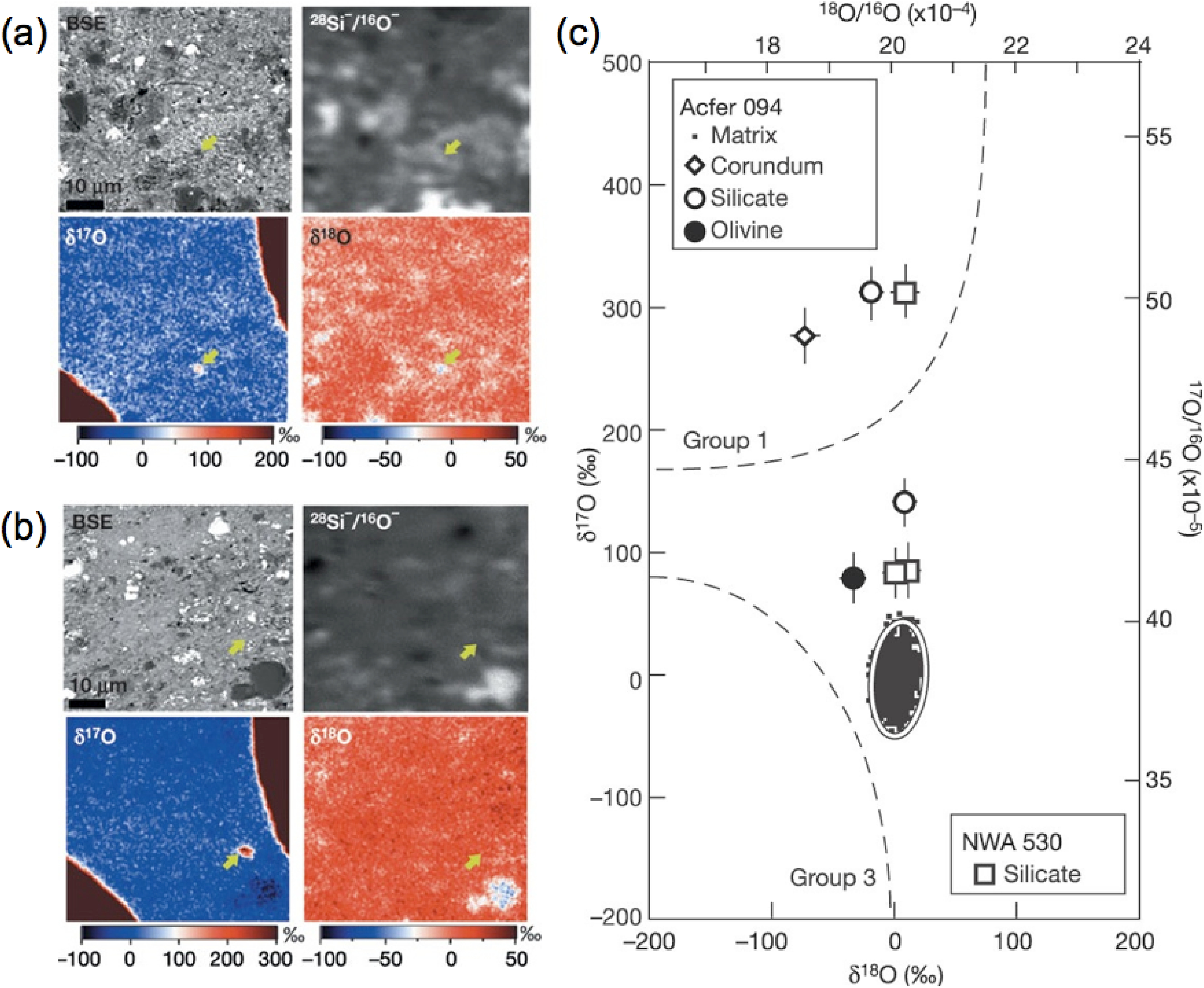
Fig. 15. Isotopic imaging of pre-solar particles in two types of chondrites. (a) Image with Acfer 094. (b) Image with NWA530.

Figure 15 shows an example of the first discovery of pre-solar particles in a chondrite meteorite using isotope microscopy.^28)^ Figures 15(a) and 15(b) are secondary electron images and isotope images of chondrite meteorites, termed Acfer094 and NWA530, respectively. Chondrite meteorites consist of tens of microns to several millimeters in diameter and have a matrix of fine particles <5 μm in diameter. The matrix is considered to be the primitive material of the solar system, which escaped the heating process in the primordial solar nebula; therefore, a pre-solar particle search was conducted in the matrix. The secondary electron image shows the detailed microstructure and composition of the sample surface, while the oxygen isotope ratio image (δ ^17^O, δ ^18^O) by MSI reveals something completely invisible by the conventional method. In the oxygen isotope ratio image, a spot (arrow) showing a completely different isotope ratio from that of the surrounding area can be observed in the almost uniform oxygen isotope ratio (the average value in the solar system). A plot of δ^17^O and δ^18^O from the obtained oxygen isotope ratio image is shown in Fig. 15(c). The isotopic composition of the spot is extremely different from that of the matrix (the region indicated by the ellipse: the average value of the solar system), indicating an isotopic anomaly. The isotopic anomaly is thus discovered, and the pre-solar particles are investigated in detail.

(Upper left) Reflection electron image by scanning electron microscopy. (Upper right) Secondary ion ratio image (^28^Si^−^/^16^O^−^). Oxygen isotope ratio images; (lower left) ^17^O/^16^O, (lower right) ^18^O/^16^O (values are based on oxygen isotope ratios in the matrix and deviations from the standard are expressed in thousandths). (c) Oxygen isotope ratio plots of pre-solar particles and matrix (an aggregate of submicron-sized fine particles) in chondrites. The pre-solar particles exhibit very different oxygen isotope ratios from those of the matrix. (Reproduced with permission from Ref. 28. ©Nature Publishing Group, 2004).

MSI will enable us to identify the location of pre-solar particles on the sample surface, and to visualize the mineral composition and surrounding microstructure of the pre-solar particles, which will facilitate a detailed investigation into the creation of the solar system and eventually the galaxy. The isotope MSI with its high precision and high spatial resolution can be applied in space science and other fields such as earth science, environmental science, life science, medicine, agriculture, archaeology, humanities, and social science.

## 4. VARIOUS TECHNOLOGIES IN MSI

In the past, various applications of MSI have been discussed, and various techniques have been developed and employed. In particular, numerous ionization methods have been applied to MSI and have proved be effective. In this section, I discuss a technique for improving the spatial resolution of laser imaging and a technique for imaging a live sample by ionization under atmospheric pressure.

### (1) MSI with inorganic nano-particle matrix^22)^

**Figure 16:**
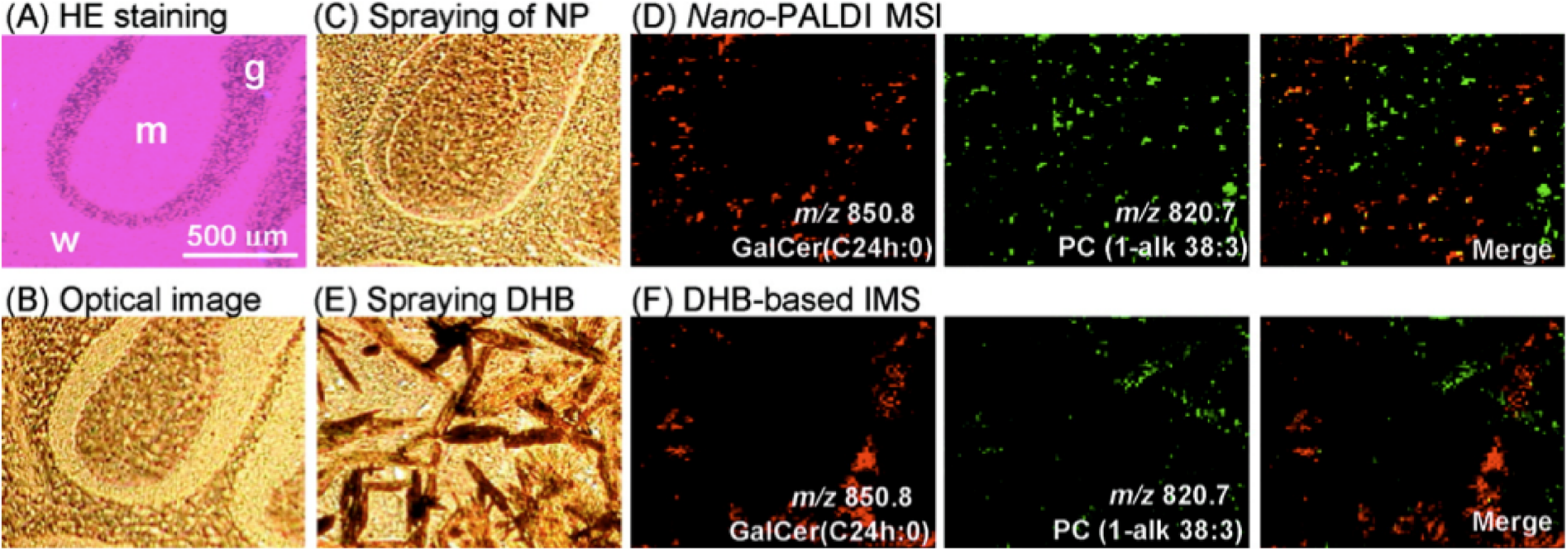
Fig. 16. Imaging using microparticle matrix. (a) Mouse cerebellar imaging area-stained image. (g: granular layer, m: molecular layer, w: white matter). (b) Optical image. (c) Image of the surface of the sample after the delivery of microparticles. (d) Imaging results of glycolipid (*m*/*z* 850) and phospholipid (*m*/*z* 827) by microparticles. (e) Surface image of the sample with 2,5-dihydroxybenzoic acid (2,5-DHB) as a matrix. (f) *m*/*z* 850 and 827 imaging results of 2,5-DHB. (Reproduced with permission from ref. 22. ©American Chemical Society, 2008).

In the imaging of a small area (∼2 mm square) using MALDI, the state of the matrix crystal sometimes affects the image obtained. In particular, the effect of the matrix state on the images obtained by MALDI is remarkable in the case of 2,5-dihydroxybenzoic acid (2,5-DHB), which forms needle-like crystals. In order to solve this problem, an imaging method using nanoparticle-assisted laser desorption ionization (nano-PALDI) has been developed. The fact that cobalt particles mixed with glycerol can ionize proteins^29)^ indicates that the particles function as a matrix. Taira *et al.*^22)^ developed functional nano-particles consisting of an iron core covered with silicon dioxide (SiO_2_) with hydroxyl and amino groups are used as the matrix. The diameter of the nano-particles is 3.7 nm, and they are dispersed in a solvent and sprayed on the sample surface. Unlike ordinary matrices, the nano-particles do not crystallize, and the structure of the tissue surface can be observed, even after the matrix is supplied, as shown in Figs. 16(b) and 16(c). On the other hand, after supplying 2,5-DHB, observation is not possible due to the formation of needle-like crystals on the tissue surface. Figures 16(d) and 16(f) show the distributions of *m*/*z* 850 (galactosyl ceramide) and 820 (phosphatidyl choline) obtained by each method. The imaging area is 1.5×1.0 mm^2^. Nano-PALDI imaging shows that *m*/*z* 850 is localized in the granular layer (g), and *m*/*z* 820 is distributed in the molecular layer (m) and white matter (w). On the other hand, in MALDI imaging, the distribution of each molecule does not differ significantly, but the crystal shape of the matrix is reflected in the imaging image. In the imaging of such a small region, particularly in projection-type imaging where the image is magnified by ion optics by irradiating a uniformly spread laser to the small region, it is considered necessary to use fine particles as the matrix.

As previously explained in “Application in pharmacokinetics,” in the case of MALDI, matrix-derived peaks in the low-molecular-weight region can be adulterants of the target peak. Since the nano-particles themselves are not ionized by PALDI, the matrix-derived peak is not detected. Therefore, PALDI is expected to be a powerful tool for imaging low-molecular-weight materials.

### (2) Application of atmospheric pressure ion source to MSI

**Figure 17:**
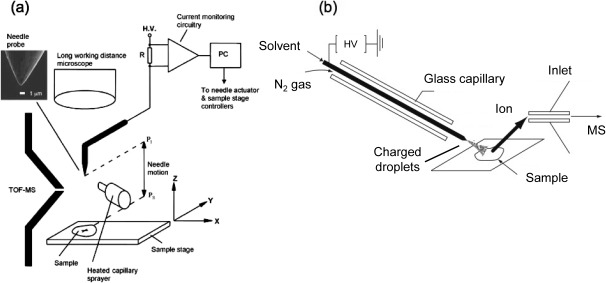
Fig. 17. (a) Probe electrospray ionization (PESI). (b) Schematic diagram of desorption electrospray ionization (DESI). (Figure (a) is reproduced with permission from Ref. 30 ©John Wiley & Sons, Ltd., 2010).

**Figure 18:**
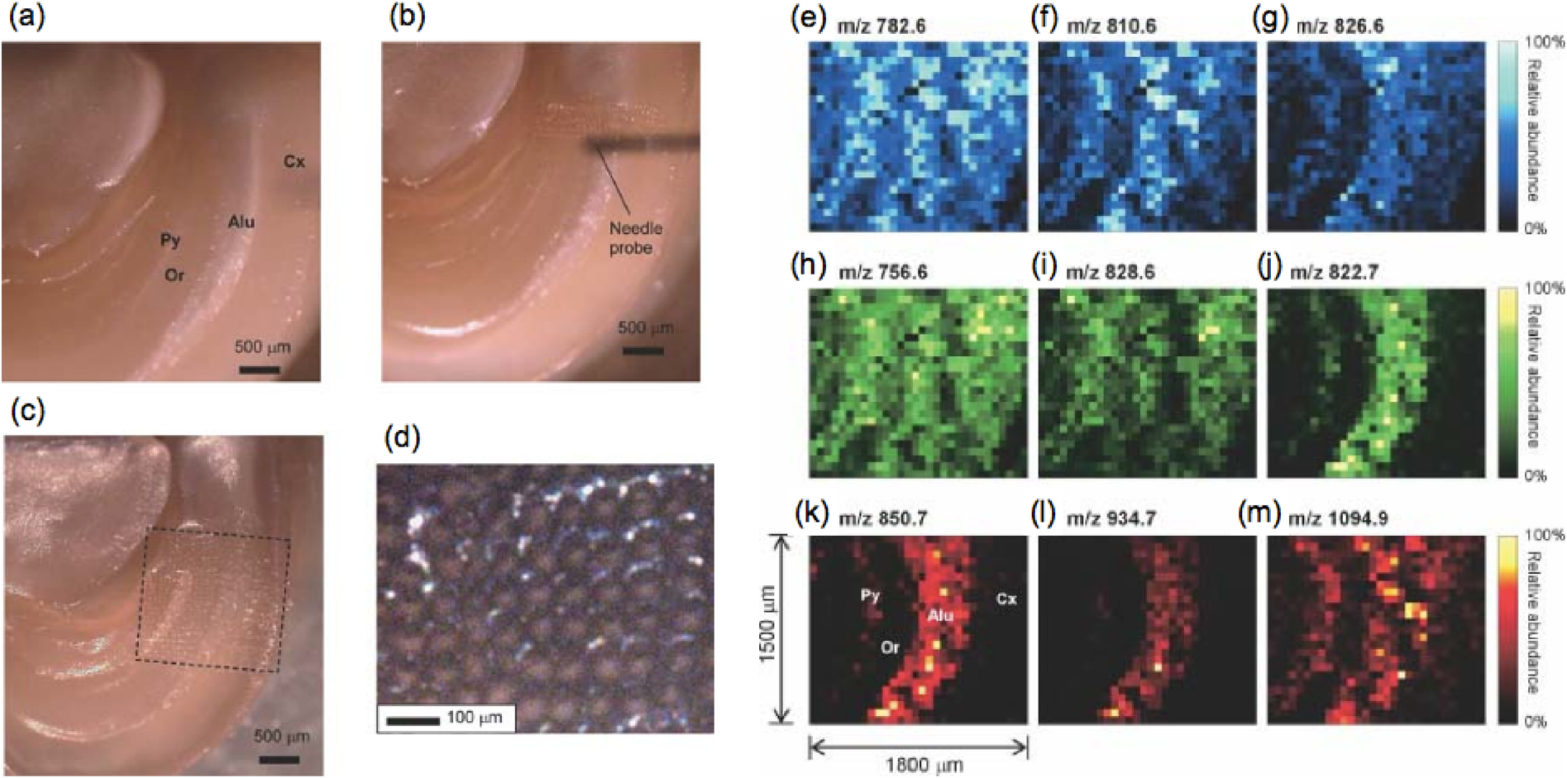
Fig. 18. Mouse brain imaging results by PESI. (a)–(c) Optical images of the captured area (Cx; cerebral cortex, Alu; hippocampal white plate, Or; ascending layer, Py; pyramidal cell layer). (d) Tissue surface photograph after imaging. (e)–(m) Imaging results at various *m*/*z*. (Reproduced with permission from Ref. 30. ©John Wiley & Sons, Ltd., 2010).

In the imaging results discussed thus far, the pretreated sample is introduced into the ion source and ionized under vacuum to obtain the ion image. In recent years, the development of ion sources capable of ionization under atmospheric pressure has been a very active field in mass spectrometry. Currently, various types of atmospheric pressure ion sources have been developed and applied to low or non-invasive imaging without pretreatment. In this article, we will focus on imaging using probe electrospray ionization (PESI)^30)^ and desorption electrospray ionization (DESI).^31)^ The schematic diagram of each ion source is shown in Fig. 17. In PESI (Fig. 17(a)), the biomolecules in the sample are attached to the surface of the probe by inserting a very fine needle (1 μm tip) on the tissue surface (Figs. 18(a), 18(b)). A DC high voltage is applied to the tip of the probe, which generates an electrospray at the tip and ionizes the sample. As shown in Figs. 18(c) and 18(d), regular sampling traces are visible after imaging. The resolution of the imaging depends on the sampling interval. Figures 18(e–l) show the imaging results of the region shown in Fig. 18(a), and site-specific distributions of phospholipids and glycolipids were obtained. As for PESI, the scanning probe electrospray ionization (SPESI) ion source has recently been introduced.^31)^

**Figure 19:**
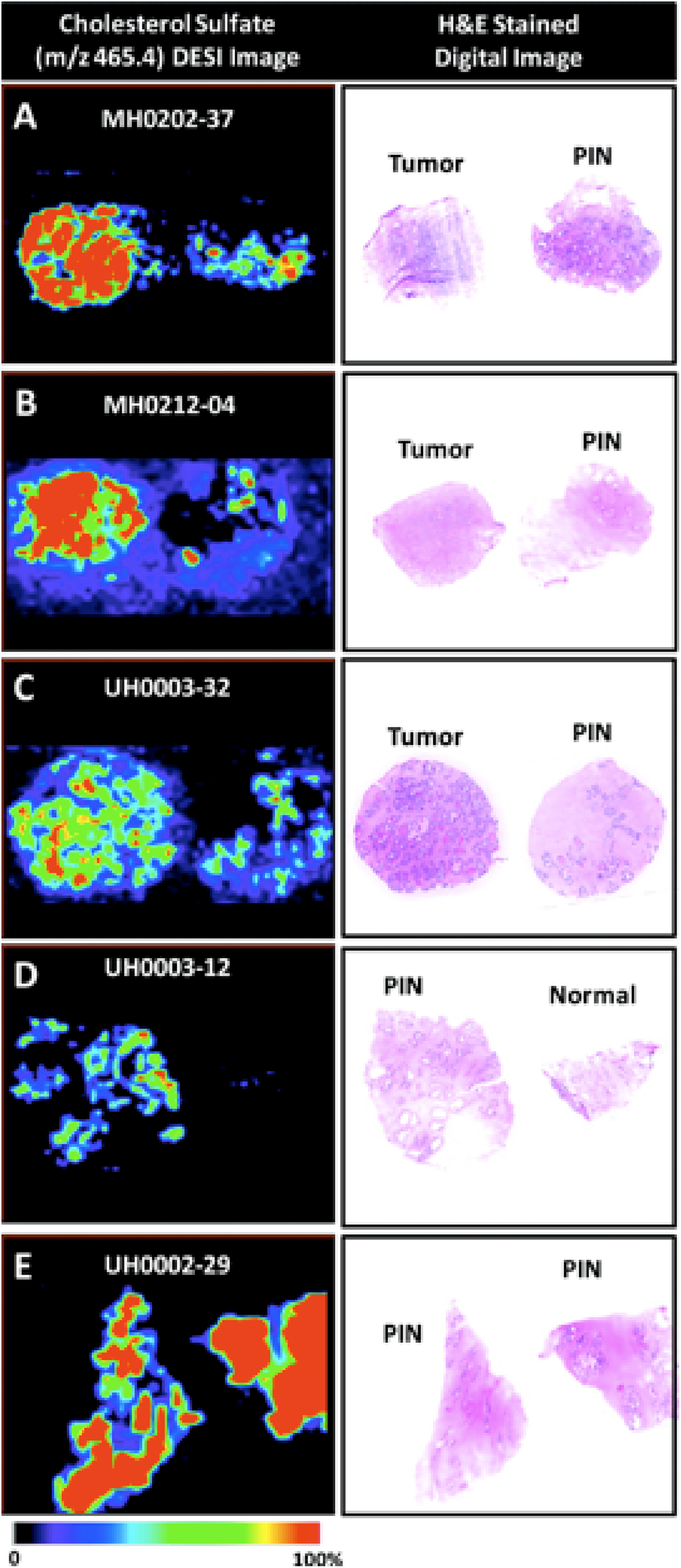
Fig. 19. Example of sulfated cholesterol (*m*/*z* 465) imaging in pathological tissue by DESI. (Reproduced with permission from Ref. 32. ©American Chemical Society, 2010).

On the other hand, DESI, shown in Fig. 17(b), is an ionization technique in which the electrospray is directly injected onto the tissue surface. Like PESI, DESI can be performed under atmospheric pressure. Compared with PESI, in which the sampling is performed with a needle, electrospray is performed by spraying a solvent (*e.g.*, acetonitrile, acetic acid, and alcohol) on the tissue surface, thus almost enabling non-invasive imaging. Although the spatial resolution of the imaging is low because the ionization is performed by spraying the solvent, the imaging results of tumor, prostate intraepithelial tumor (PIN), and normal tissue (normal) shown in Fig. 19 indicate that tumor- and PIN-specific sulfated cholesterol (*m*/*z* 465.4) was detected specifically in tumor and PIN.^32)^

In MSI using an atmospheric pressure ion source, it is possible to measure raw samples without pretreatment in a low and non-invasive manner. In fact, the example of PESI shown in Fig. 18 is based on a small section of mouse brain (7×5×2 mm^3^). It is expected that the measurement method using an atmospheric pressure ion source will be used in the medical field in the future.

## 5. SUMMARY

Here, I reviewed the methodology of MSI, its applications in various fields, and new techniques. MSI is becoming a powerful tool in terms of label-free, multi-component, and specific visualization. In addition, high-resolution imaging and imaging under atmospheric pressure are becoming a reality. It is expected that MSI will be used for research purposes as well as in various fields, such as medicine.

## Notes

Mass Spectrom (Tokyo) 2022; 11(1): A0102
